# DETERMINANTS OF ALZHEIMER’S DISEASE AND RELATED DEMENTIA RISK IN OLDER BLACK MEN

**DOI:** 10.1093/geroni/igad104.0359

**Published:** 2023-12-21

**Authors:** Darlingtina Esiaka

**Affiliations:** University of Kentucky, Lexington, Kentucky, United States

## Abstract

Beyond biological determinants of Alzheimer’s disease and related dementias (ADRD), extensive literature also suggests a role for sociocultural determinants—including race, ethnicity, education, neighborhood, and economic environment—in modulating cognitive outcomes. In recent years, research examining sociodemographic disparities in ADRD incidence points to modifiable mechanisms. Important for more personalized dementia prevention is the focus on how social determinants of health interact with lifestyle factors to put people at risk of developing ADRD. However, a historical challenge to research on dementia disparities and prevention relates to stark underrepresentation of Black individuals, particularly Black men. Therefore, this within-group study utilizes data from older Black men to examine social determinants (e.g., neighborhood, home environment, social cohesion), lifestyle factors (e.g., sleep, diet, drinking), and their impact on neurocognitive domains linked to ADRD. Results from 178 Black men, age 70+, from the 2021 National Health and Aging Trends Study show lower presence of neighborhood physical disorders such as vandalism (B=.064, p=0.035) and better sleep quality (B=.022, p=0.036) were associated with better performance on executive functioning tasks. Additionally, ongoing primary data collection (N=100 Black men, age 60+) supports these findings and provides additional insights toward identifying and validating risk/protective factors that predict cognitive outcomes very early in the cognitive aging process among Black men. This work represents a necessary step toward a better understanding of heightened ADRD risk in this understudied group. Results can be used to guide tailored interventions likely to be most effective for mitigating risk and slowing progression in Black men.

